# Ubiquitination in Periodontal Disease: A Review

**DOI:** 10.3390/ijms18071476

**Published:** 2017-07-10

**Authors:** Sachio Tsuchida, Mamoru Satoh, Masaki Takiwaki, Fumio Nomura

**Affiliations:** 1 Division of Clinical Mass Spectrometry, Chiba University Hospital, 1-8-1 Inohana, Chuo-ku, Chiba 260-8670, Japan; msatoh1995@yahoo.co.jp (M.S.); takiwaki@chiba-u.jp (M.T.); fnomura@faculty.chiba-u.jp (F.N.); 2 Division of Laboratory Medicine, Clinical Genetics and Proteomics, Chiba University Hospital, 1-8-1 Inohana, Chuo-ku, Chiba 260-8670, Japan

**Keywords:** ubiquitination, proteasome, periodontal diseases

## Abstract

Periodontal disease (periodontitis) is a chronic inflammatory condition initiated by microbial infection that leads to gingival tissue destruction and alveolar bone resorption. The periodontal tissue’s response to dental plaque is characterized by the accumulation of polymorphonuclear leukocytes, macrophages, and lymphocytes, all of which release inflammatory mediators and cytokines to orchestrate the immunopathogenesis of periodontal disease. Ubiquitination is achieved by a mechanism that involves a number of factors, including an ubiquitin-activating enzyme, ubiquitin-conjugating enzyme, and ubiquitin–protein ligase. Ubiquitination is a post-translational modification restricted to eukaryotes that are involved in essential host processes. The ubiquitin system has been implicated in the immune response, development, and programmed cell death. Increasing numbers of recent reports have provided evidence that many approaches are delivering promising reports for discovering the relationship between ubiquitination and periodontal disease. The scope of this review was to investigate recent progress in the discovery of ubiquitinated protein in diseased periodontium and to discuss the ubiquitination process in periodontal diseases.

## 1. Introduction

The investing and supporting structures of the teeth consist of the attachment apparatus, which includes the cementum of the root, periodontal ligament (PDL), alveolar process, gingiva, and mucous membrane covering [1,2,3,4]. These tissues are collectively referred to as the periodontium [1,2,3,4]. Periodontal disease is a bacterial infection that destroys the gingiva and its surrounding tissues [5]. The most common causative bacteria for periodontal disease are gram-negative anaerobic or aerobic species, such as *Porphyromonas gingivalis*, *Prevotella intermedia*, and *Fusobacterium nucleatum* [6,7,8].

Ubiquitination is a post-translational modification restricted to eukaryotes and is involved in essential host processes; it is achieved by a mechanism that involves a number of factors, including a ubiquitin-activating enzyme (E1), a ubiquitin-conjugating enzyme (E2), and a ubiquitin–protein ligase (E3) [9]. The ubiquitin system has been implicated in the host immune response, development, and programmed cell death [10].

A recent study suggested that polyubiquitinated proteins accumulate on the bacterial surface, a response that has been confirmed in several cell types [11]. The ubiquitin system plays a major role in recognition of bacterial pathogens in the cytosol of mammalian cells [11,12]. Kieffer et al., showed that ubiquitin N- and C-terminal-derived peptides are important for antimicrobial activity [13].

Autophagy has been described as an immune mechanism and has been implicated in the pathogenesis and host response to many diseases. Ubiquitin-adaptor proteins, such as p62, bind to ubiquitin to target antibacterial peptide precursors, and ubiquitin-like proteins mediate the selective autophagy of pathogens [14,15].

Mass spectrometry technology has emerged as an important method for identification of various post-translational modifications, including ubiquitination [16]. Proteolysis and detection using mass spectrometry is presently the analytical method of choice for studies of ubiquitinated proteins [16]. Danielsen et al., identified 5756 putative ubiquitinated proteins in human U2OS osteosarcoma cells and HEK293T embryonic kidney cells using mass spectrometric analysis [9].

Increasing numbers of recent reports have provided evidence that many approaches are delivering promising reports for discovering the relationship between ubiquitination and periodontal disease. We reviewed the scope of ubiquitination in periodontal diseases.

## 2. Periodontal Disease

**Figure 1:**
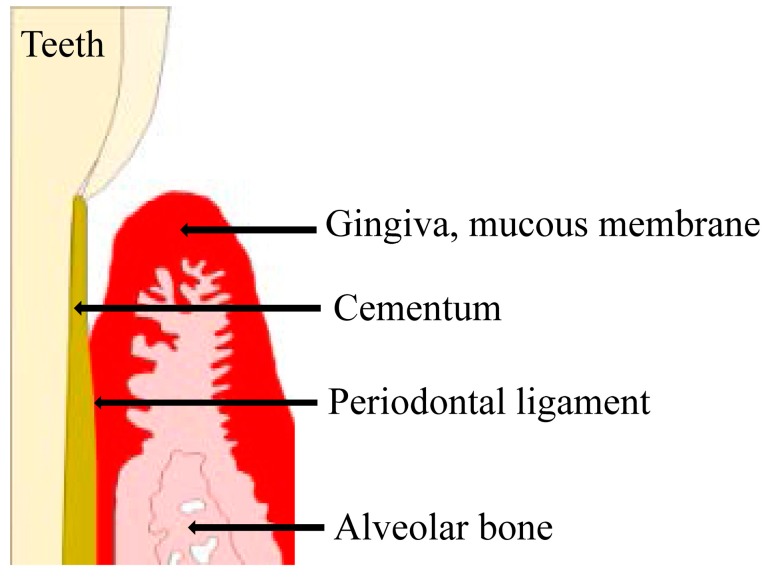
The investing and supporting structures of the teeth consist of the attachment apparatus, which includes the root cementum, periodontal ligament, alveolar process, gingiva, and mucous membrane covering. These tissues are collectively referred to as the periodontium.

Periodontal diseases are bacterial infections affecting the periodontium that result in the loss of tooth support and are associated with bacteria-induced inflammation and activation of the host immune response (Figure 1). Periodontal diseases can be considered separately, as gingivitis and periodontitis. Gingivitis refers to periodontal disease characterized by inflammation of the gums, due to excessive plaque on the teeth, without attachment loss [17]. Signs of gingivitis include red, swollen gums, or gums that bleed easily when brush teeth. Gingivitis is prevalent at all ages of dentate population and is considered to the most common form of periodontal disease. Gingival inflammation may have different causes than routine gingivitis, associated with plaque. Plaque-induced gingivitis is characterized by inflammation of the gingiva, resulting from bacteria located at the gingival margin. Non-plaque gingivitis often exhibits characteristic clinical features and can be caused by allergic reactions to dental restorative materials, toothpastes, mouthwashes, or foods [18].

Periodontal disease is characterized by the destruction of the hard tissue and soft connective tissue constituents of the periodontium. Periodontitis is a chronic infectious disease of the tissues surrounding the teeth, caused by microorganisms. Its key features include periodontal pocket formation, loss of connective tissue attachment, alveolar bone resorption, and gingival inflammation. Left untreated, these processes frequently result in tooth loss. One key sign of periodontitis is pulling away of the gingival (gum) tissue, from the teeth. This process creates pockets where additional bacteria can build up, potentially causing an infection. Periodontal diseases range from simple gingival (gum) inflammation at the gingival margin of the teeth (gingivitis), to serious irreversible damage to the soft gingival tissue and alveolar bone that supports the teeth (periodontitis) [19,20]. Mild periodontitis is the earliest form and occurs when plaque begins to harden into a biofilm which combines with the host response and systemic parameters (such as diabetes and smoking et cetera) to form calculus in the space between the gums and teeth.

There are seven major categories of destructive periodontal disease: (1) chronic periodontitis; (2) localized aggressive periodontitis; (3) generalized aggressive periodontitis; (4) periodontitis as a manifestation of systemic disease; (5) necrotizing ulcerative gingivitis/periodontitis; (6) abscesses of the periodontium; and (7) combined periodontic–endodontic lesions as per the 1999 periodontal disease and condition classification system [19,20]. The diagnosis of periodontal disease is based on a detailed clinical examination, medical and dental histories, degree of tooth mobility, and radiographic assessment. Primary clinical measurements (probing pocket depth, bleeding on probing, clinical attachment loss, plaque index, and X-ray findings) used for periodontal disease diagnosis are indicators of a previous periodontal disease rather than the present disease activity; thus, they have limited utility. Nonetheless, these clinical parameters provide important information about the level of tissue destruction.

The periodontal pocket exhibits increased depth either because of the inflammatory swelling of the marginal gingiva in a coronal direction or because of the destruction of the fiber attachment to the cementum apical to the sulcus, resulting in separation of the gingival tissues from the tooth. Periodontitis occurs in three progressive stages: mild, moderate, and severe. In mild periodontitis, the probing depth of the gingival crevice ranges from 4 to 5 mm; in moderate periodontitis, it ranges from 6 to 7 mm, and in severe periodontitis it is ≥8 mm [19,21].

In the conventional periodontal treatment, mechanical tools such as hand curettes and power-driven scalers are mainly used for subgingival debridement in periodontal pockets to arrest the disease. However, such mechanical therapy is sometimes insufficient for a complete removal of bacterial and calcified deposits from the diseased root surface. Therefore, bacteria remaining in the periodontal pockets may eventually cause disease recurrence [22]. The modern era of periodontal research began in the mid and late 1960s with documentation of the fact that gingivitis and periodontitis in humans are caused by bacteria. The recognition and diagnosis of periodontal disease involves an awareness of the clinical and biological processes of the histological changes that occur in affected tissues. An understanding of these criteria is fundamental to the prevention and treatment of periodontal disease and to the maintenance of oral health.

Recent studies have reported a definite association between periodontal disease and other inflammatory conditions of the body. Interestingly, periodontal disease increases the risk of stroke, heart disease, diabetes, pulmonary problems, and other serious systemic diseases [23,24,25]. Diabetes mellitus (DM), a systemic disease with several major complications affecting both the quality and length of life, is known to modify periodontal disease expression by inducing a hyper inflammatory host response to bacterial challenge [26,27]. Different pathophysiological mechanisms have been suggested for periodontal disease susceptibility among patients with DM [26,28].

For a long time, it was thought that bacterial infection per se was the primary factor that linked periodontal disease to systemic disease. However, recent studies have demonstrated that inflammation evoked by bacterial infection may be responsible for this association [28].

## 3. Bacterial Pathogens of Periodontal Disease

Bacterial pathogens have been strongly associated with the onset and progression of periodontal disease. A consideration of some of the more general aspects of microbial dental plaques, i.e., development and heterogeneity, microbial succession, composition and structure, and mechanisms of formation, necessarily precedes a discussion of the microbial composition of the gingival crevice (subgingival plaque). Microbial dental plaques may be considered as heterogeneous, dense, non-calcified, bacterial masses intimately associated with the tooth surface and usually so firmly adhered to the surface that they are not washed off by salivary flow. Some varieties of subgingival plaque are nonadherent and consist of large numbers of motile organisms.

Socransky et al., examined over 13,000 subgingival plaque samples, from 185 adult subjects. Using DNA hybridization, the authors confirmed the presence of specific microbial groups within dental plaque [29]. The authors used cluster analysis and community ordination techniques to examine the relationships among bacterial species.

Two complexes consist of microorganisms thought to be the major etiologic agents of periodontal disease. One complex, called the “Red Complex,” consisted of 3 closely-related species: *Porphyromonas gingivalis*, *Tannerella forsythia*, and *Treponema denticola.* The Orange Complex consists of: *Fusobacterium nucleatum*, *Prevotella intermedia*, *Prevotella nigrescens*, *Peptostreptococcus micros*, *Streptococcus constellatus*, *Eubacterium nodatum*, *Campylobacter showae*, *Campylobacter gracilis*, and *Campylobacter rectus*.

Disease progression occurs as a result of bacterial actions on host tissues, as well as from self-damaging host responses to the colonizing bacteria. While no single species has been implicated as the primary pathogen, and available evidence appears consistent with a polymicrobial etiology, the Red Complex bacteria consisting of *P. gingivalis*, *T. denticola* and *Tannerella forsythia* are implicated in periodontitis.

*Treponemes* cause several chronic human diseases including syphilis, yaws (*Treponema pallidum*), periodontal diseases (including chronic periodontitis), acute necrotizing ulcerative gingivitis (*T. denticola*, *T. lecithinolyticum*, *T. socranskii*, and others), endodontic infections, and some acute dental abscesses [30,31,32]. Basic research and clinical evidence suggests that the prevalence of *T. denticola*, together with high numbers of other proteolytic gram-negative bacteria in periodontal pockets, may assist periodontal disease progression [33,34]. Among periodontal anaerobic pathogens, the oral spirochetes, particulalry *T. denticola*, are associated with periodontal diseases such as early-onset periodontitis. *T. denticola* adheres to fibroblasts and epithelial cells, as well as to extracellular matrix components present in periodontal tissues, producing several factors that may increase bacterial virulence [33,34].

A member of the Orange Complex, *P. intermedia* is a short, rounded, nonmotile gram-negative anaerobic pathogenic rod which is less virulent, and less proteolytic than *P. gingivalis* [29]. It is one of the major periodontal pathogens, possessing various virulence factors such as hemolysin, hemagglutinin, proteolytic and hydrolytic enzymes, allowing it to colonize in the oral cavity, evade host defenses, modulate immune responses, and cause tissue destruction [35]. *P. intermedia* can induce pro-MMP-2 and pro-MMP-9 expression in fetal mouse osteoblasts [36]. *P. intermedia* appears associated with advanced periodontitis, and *P. nigrescens* predominates in healthy gingiva, in children [37,38]. We found that both *P. intermedia* and *P. nigrescens* were predominant in adults and children who had subclinical, early to moderate-stage periodontal disease, for which no treatment but tooth brushing was required. The development and progression of periodontal disease have been associated with specific gram-negative bacteria in subgingival plaque, especially *F. nucleatum* and *P. gingivalis* [7,39]. Furthermore, these two are the most common bacteria encountered in extraradicular biofilms covering the apices of the roots in refractory apical periodontal disease.

Bacterial coaggregation is a key mechanism in biofilm formation and has been extensively studied in the context of dental plaque formation and periodontal disease. Several studies investigating the dental biofilm formation have reported that *F. nucleatum* is a key component in this process, serving both as an early colonizer and a “bridge organism” that facilitates colonization of other bacteria [40,41,42]. *F. nucleatum* is a periodontal pathogen associated with a wide spectrum of human diseases. It is ubiquitous in the oral cavity but absent or infrequently detected elsewhere in the body under normal conditions [43]. This bacterium is very much associated with periodontitis, along with invasive human infections of the head and neck, chest, lung, liver, etc. Furthermore, when isolated from human intestinal biopsy material, *F. nucleatum* has been found to be more readily culturable from patients with a gastrointestinal disease than from healthy controls, and the strains grown from the inflamed biopsy tissue tend to display a more invasive phenotype [44]. Due to its adherence ability, *F. nucleatum* can be associated with viruses that adhere to host tissue cells as an invasion process. The primary role of *F. nucleatum* in the etiology of periodontal disease is to provide a sub-gingival habitat to promote the proliferation and colonization of more aggressively virulent organisms such as *P. gingivalis.*

Periodontal disease is an inflammatory lesion initiated by gram-negative periodontal bacterial pathogens such as *P. gingivalis*. Periodontal pathogens destroy the tissue primarily through the production of proteolytic enzymes. *P. gingivalis* produces at least 10 proteolytic enzymes, of which the studied are the gingipains: the Arg-X-specific enzymes RgpA and RgpB and the Lys-X-specific enzyme Kgp, which comprise a family of isoforms that can be post-translationally modified with glycan chains and that can be presented on the cell surface or secreted into the extracellular milieu [45]. These enzymes degrade the structural components of the periodontium, along with immune effectors, such as antibodies, cytokines, complement components, and associated receptors. *P. gingivalis*, when shed from the biofilm, can invade and survive within gingival epithelial cells [46]. Epithelial cells with internalized *P. gingivalis* are resistant to apoptotic death and indeed accelerate through the cell cycle [47]. Previous studies have demonstrated that lipopolysaccharide (LPS) from periodontal pathogens stimulates host cells to secrete proinflammatory mediators such as interleukin (IL)-1, IL-6, tumor necrosis factor-α (TNF-α), and prostaglandin E2 [48,49,50].

*P. gingivalis* has been implicated as an accessory factor in certain systemic conditions, such as atherosclerotic heart disease and aspiration pneumonia [28].

Hence, this pathogen is the most intensively studied oral organism at the molecular level, and its pathogenicity is attributed to a panel of potential virulence factors, such as cysteine protease and LPS. The LPS molecules that comprise the outer leaflet of the outer membrane of gram-negative bacteria are representative of microbe-associated molecular patterns and possess diverse biological activities. LPS from periodontal pathogens stimulates host cells to induce production of proinflammatory mediators such as IL-1, TNF-α, IL-6, and prostaglandin E2, which in turn, induce receptor activator of nuclear factor κB ligand (RANKL), a critical osteoclast differentiation factor, in osteoblasts [28,37,39]. LPS that is either bound to or released from bacterial cells serves as an activator of the host innate immune response by stimulating the expression of proinflammatory cytokines, which are required for development of a local inflammatory response to bacterial infection.

## 4. Ubiquitination in Periodontal Disease

Ubiquitination is essential to cell growth and viability and is important in cell cycle regulation, DNA repair, morphogenesis, signaling, and immune function [51]. Recently, the research on ubiquitination and its contribution to periodontal disease has been progressing steadily (Table 1).

Maekawa et al., described *P. gingivalis* (Pg)-co-activation of Toll-like receptor 2 (TLR2) and C5a receptor (C5aR) in neutorophils [52]. The resulting crosstalk leads to ubiqutination and proteasomal degradation of MyD88, thereby inhibiting a host-protective antimicrobial response. This activity requires C5aR/TLR2-dependent release of transforming growth factor-β1, which mediates ubiquitin–proteasome degradation of MyD88, via the E3 ubiqutin ligase Smad ubiquitin regulatory factor 1. MyD88 is unlikely to contribute to immune evasion mediated by the *P.g*-induced C5a-TLR2 crosstalk; however it does contribute to the clearance of *P.g.* infection [52].

Cai et al., proposed that muramyl dipeptide (MDP) highly activated signaling pathways in response to *P. gingivalis* infection [53]. *P. gingivalis*-induced TNF-α expression can be affected by MDP in a biphasic, concentration-dependent manner. MDP is transferred into the cytoplasm, where it activates c-Jun N-terminal kinases (JNKs), which up-regulate activator protein-1 (API). JNKs are essential regulators of physiological and pathological processes in several diseases. API activates the editing ubiquitin enzyme A20 and restricts ubiquitination of nucleotide-binding oligomerization domain-containing protein 2, consequently inhibiting TNF-α secretion in response to *P. gingivalis* infection.

A20 is a potent inhibitor of NF-κB signaling [54]. The ubiquitin-editing protein A20 has been shown to negatively regulate NF-κB signaling by multiple mechanisms, such as binding of the inflammatory molecules TNF-α, IL-1β, LPS, CD40, and IL-17 to their respective cell surface receptors, which promotes the recruitment of specific adaptor proteins. A20 has also been shown to regulate autophagy triggered by the LPS-receptor Toll-like receptor 4 [55]. Hong YJ et al., evaluated the effects of A20 overexpression on the inflammatory response in patients with periodontitis and found that A20 was upregulated in the gingival tissues and neutrophils and in LPS-exposed human PDL cells, suggesting that A20 overexpression may be a potential therapeutic target in inflammatory bone loss diseases, such as periodontal disease [56].

Tsuchida et al., identified the antimicrobial peptide dermcidin (DCD) in the gingival crevicular fluid (GCF) using proteomic analyses [57]. Moreover, Western blot analysis revealed that the molecular weights of GCF protein bands considerably varied (approximately 27 kDa) [57]. This group also attempted to explore the considerable variation in the molecular weights of protein bands using on-membrane digestion and liquid chromatography–tandem mass spectrometry analyses. In a more recent study, Tsuchida et al., examined the role of ubiquitin among DCD-interacting proteins in immunoprecipitation experiments and detected ubiquitinated DCD using Western blotting and immunoprecipitation with antibodies against DCD and mono-/poly-ubiquitinated proteins [58]. These analyses indicated the possible involvement of DCD in the ubiquitination process [58].

Gosh et al., found that a disease-associated fibronectin fragment triggers apoptosis of primary human PDL cells via novel apoptotic pathway in which the tumor suppressor p53 is transcriptionally downregulated [59]. Moreover, this group investigated whether fibronectin fragments induce ubiquitination of p53 and its degradation through the proteasome [59]. Inhibition of either the proteolytic function of the proteasome or suppression of ubiquitin expression at the protein level prevented degradation of p53 and subsequent apoptosis of primary PDL cells [59]. These findings provide potential therapeutic targets and treatment strategies for inflammatory diseases such as arthritis and periodontal disease.

Li et al., evaluated the activation status of autophagy in keratocystic odontogenic tumors (KCOT) and detected and compared the expression patterns of some key autophagy-related proteins in clinical samples of KCOT and radicular cysts [60]. The results showed that both mRNA expression and immunoreactivity of autophagy-related proteins were considerably increased in KCOT compared with radicular cysts, implicating autophagy activation in KCOT and showing its possible association with growth potential [60].

Zeidán-Chuliá et al., generated a protein–protein network model of interaction using the STRING 10 database, a search tool for the retrieval of interacting genes/proteins, and found that ubiquitin C (UBC), Jun proto-oncogene (JUN), and matrix metalloproteinase-14 (MMP-14) were most involved in central hub- and non-hub-bottlenecks among the 211 genes/proteins of the whole interactome [61]. This group also reported that UBC, JUN, and MMP-14 were likely optimal candidate host-derived biomarkers, in combination with oral pathogenic bacteria-derived proteins, for detection of periodontitis in the early phase [61].

Upon activation of the NF-κB pathway, IκB proteins are phosphorylated by the macromolecular IκB kinase complex (IKK), which contains two catalytic subunits (IKKa and IKKb), and subsequently degraded by the ubiquitin-mediated proteasomal degradation pathway. Periodontal disease could result in destruction of PDL and alveolar bone. Upregulation of RANKL mRNA in both inflammatory cells and the epithelium has been associated with osteoclastic bone destruction in periodontal disease. Also, NF-κB activation was observed in oral epithelial cells exposed to the periodontal pathogens *F. nucleatum* and *P. gingivalis* [62]. Moreover, abnormal activation of NF-κB signaling in osteoclasts has been associated with excessive osteoclastic activity and frequently observed under osteolytic conditions, including periodontitis [62].

White sponge nevus (WSN) is a rare periodontal hereditary disease. Cai et al., investigated its pathogenesis using expression profiling and found that the ribosome structure was damaged and the translation rate was limited in WSN patients, while ubiquitin-mediated proteolysis was enhanced [63]. Their study concluded that the abnormal degradation of keratin 13 protein in WSN patients may be associated with keratin 7 protein and an abnormal ubiquitination process [63].

The proteasome is a muiltilsubunit enzyme complex that plays a vital role in the regulation of apoptosis and cell cycle progression. Before a protein is degraded, it is first flagged for destruction by the ubiquitin conjugation system, which ultimately results in the attachment of a polyubiquitinated chain to the target protein [64].

Bortezomib (BTZ) was the first proteasome inhibitor for clinical treatment of malignancies. The anti-cancer activity of BTZ is accompanied by an anti-inflammatory effect. Jiang et al., reported that in an LPS- and ligature-induced periodontal disease rat model, BTZ suppressed the expression of TNF-α, IL-1β, IL-6, and IL-8; reduced the ratio of receptor activation of RANKL/osteoprotegerin; and prevented alveolar bone resorption, suggesting that the anti-inflammatory activity of BTZ has a promising therapeutic effect against periodontal inflammatory responses in periodontal disease [65]. Kitagaki et al., investigated whether BTZ can induce differentiation of PDL cells into hard tissue-forming cells and found that BTZ enhanced the expression of bone morphogenetic protein-2, which induces cytodifferentiation and mineralization of PDL cells [66]. BTZ induced cytodifferentiation of PDL cells by enhancing the accumulation of B-catenin within the cytosol and nucleus, suggesting that BTZ may be efficacious for use in periodontal regeneration therapy [66].

*F. nucleatum* and its cell wall extracts induce expression of human β-defensin-2 (hBD2), an antimicrobial and immunomodulatory peptide, in cultured primary human gingival epithelial cells in vitro. *F. nucleatum* cell wall extracts upregulated the expression of multiple protease inhibitors and suppressed NF-κB function and the ubiquitin/proteasome system. Yin et al., reported that both *F. nucleatum* cell wall extracts and hBD2 up-regulate genes that may enhance the gingival epithelia barrier [67]. The NF-κB family of transcription factors regulates the expression of a large array of genes involved in diverse cellular processes, including inflammation, immunity, and survival. Activation of NF-κB requires ubiquitination, a highly conserved and versatile modification that can regulate cell signaling through both proteasome-dependent and -independent mechanisms [68].

Streptozotocin (STZ, 2-deoxy-2-3(3-(methyl-3-nitrosoureid)-d-glucopyranose) is an antibiotic extracted from *Streptomyces achromogenes*, a chemical agent widely used to induce diabetes in experimental animals [69]. Shin et al., first demonstrated that STZ treatment significantly suppresses growth and induces apoptosis in PDL cells [69]. STZ treatment also dramatically reduces Mcl-1 (Induced myeloid leukemia cell differentiation protein) expression in a proteasome-dependent manner, thereby suppressing growth of PDL cells through the Bax/Bak apoptotic signaling pathway [69]. STZ may play an important role in inducing PDL cell apoptosis, as a potential direct inducer of periodontitis, in the STZ-induced diabetic animal [69].

## 5. Future Directions for Periodontal Research

Loss of normal homeostasis can result from many mechanisms, including infection or stress, leading to cellular dysfunction and disease. We note an increase in ubiquitinated products, throughout a variety of pathophysiologic states associated with oxidative stressors, including neurodegenerative disease1 and coronary atherogenesis [70,71]. Ubiquitin–proteasome system (UPS) dysregulation is linked to a number of inherited and acquired diseases such as cancer, diabetes, stroke, graft injection, Alzheimer’s disease, amyotropic lateral sclerosis, multiple sclerosis, asthma, inflammatory bowel disease, autoimmune thyroiditis, inflammatory arthritis, and systemic lupus erythematosus [70,71]. UPS mediates inflammatory responses through various mechanisms [72]. Volume: The most important link between the UPS and inflammation relates to NF-κB. NF-κB is a master regulator of many inflammatory cytokine genes, and its activation is mediated through the UPS. NF-κB is actively inhibited when bound to IκB. NF-κB activation follows degradation of IκB, which is dependent on ubiquination of IκB, followed by proteasomal degradation. Hence, alterations in the UPS can have profound effects on immune responses, including inflammatory cytokine regulation. Interestingly, NF-κB activation was observed in oral epithelial cells exposed to the periodontal pathogens *F. nucleatum* and *P. gingivalis* [62]. Very little is currently known about the NF-κB activation in periodontal disease, and future research is needed to clarify this process.

The ubiquitin–proteasome pathway is a therapeutic target for patients with hematological malignancies such as multiple myeloma or non-Hodgkin lymphoma. Development of BTZ, a first generation proteasome inhibitor, is guided by laboratory studies clinical drug application of this drug in these diseases. BTZ may be efficacious for use in periodontal regeneration therapy.

Systematic analysis and targeting of individual UPS components will unravel and discriminate regulatory mechanisms that contribute to, and protect against, progressive cardiac disease [73]. Integrating this knowledge with drug design may reduce cardiovascular adverse effects, as observed in patients with noncardiac diseases treated with proteasome inhibitors. Our understanding of the molecular action of ubiquitin, in signaling pathways, is increasing. Additionally, we are studying how alterations in the ubiquitin system contribute to periodontal disease development. Technological advances in the field of proteomic mass spectrometry, combined with the development of specific antibodies against Ub chains or Ub remnants on substrates, allow investigators to precisely trace ubiquitination throughout the genome. As a result, we identified novel Ub-regulatory enzymes and adaptors as possible targets for developing more selective therapeutic compounds for treatment of periodontal disease.

## 6. Conclusions

Although much remains unknown, proteasomes research on seeks to find stronger ties between the ubiquitin–proteasome system pathway and various diseases. With recent advancements in the field of cellular biology and proteasomal pathways, comes an optimistic outlook toward new, and more effective, treatments for many illnesses that affect mankind. It is therefore expected that elucidating the underlying mechanisms of ubiquitination-associated periodontal disease will help clarify these poorly understood mechanisms.

## Figures and Tables

**Table 1:** Summary of studies on ubiqutin in periodontal diseases.

Authors	Research Object (Bacteria, Cell, Fluid, or Tissues)	Methodolgy	Essence of a Discourse or Summary of Results	Reference
Maekawa, et al., 2014	*Porphyromonas gingivalis*, Human neutrophils	Innate pattern recognition of *P.g* is predominantly mediated by TLR2, which synergizes with C5aR during periodontal inflammation. The authors examined Pg interactions with both C5aR and TLR2 using knockout mice and specific inhibitors.	*Porphyromonas gingivalis* (*P.g*)-induced co-activation of toll-like receptor 2 (TLR2) and the C5a receptor (C5aR) in neutorophils; the resulting crosstalk leads to ubiqutination and proteasomal degradation of MyD88, thereby inhibiting a host-protective antimicrobial response. This activity requires the C5aR/TLR2- dependent release of transforming growth factor-β1, which mediates ubiquitin-proteasome degradation of MyD88 via the E3 ubiqutin ligase Smad ubiquitin regulatory factor 1. MyD88 is unlikely to contribute to immune evasion mediated by the *P.g*-induced C5a-TLR2 crosstalk; however, it contributes to *P.g* infection clearance.	[52]
Cai et al., 2016	*Porphyromonas gingivalis*	*P.g.*-exposed macrophages were treated with 10 μg/mL MDP (MDP-low) up-regulated TNF-α by 29%, while 100 μg/mL or higher (MDP-high) significantly decreased the level of TNF-α (16–38%).	API activates the ubiquitin-editing enzyme A20 and restricts ubiquitination of nucleotide-binding oligomerization domain-containing protein 2, consequently inhibiting TNF-α secretion in response to *P. gingivalis* infection.	[53]
Hong, et al., 2016	Gingival tissues, Human periodontal ligament cell	The concentration of prostaglandin E2 was measured by a radioimmunoassay. Reverse transcription-polymerase chain reactions and Western blot analyses were used to measure the mRNA and protein levels, respectively. Osteoclastic differentiation was assessed in mouse bone marrow-derived macrophages using conditioned medium from LPS- and nicotine-treated hPDLCs.	The ubiquitin-edting protein A20 was upregulated in the gingival tissues and neutrophils of patients with periodontal disease and in LPS-exposed human periodontal ligament cells.	[56]
Tsuchida, et al., 2016	Gingival crevicular fluid	The authors explored the considerable variation in the molecular weights of protein bands using on-membrane digestion and liquid chromatography-tandem mass spectrometry (LC–MS/MS) analyses. In immunoprecipitation experiments, ubiquitin DCD was detected by Western blotting and by immunoprecipitation.	In immunoprecipitation experiments, ubiquitinated antimicrobial peptide dermcidin (DCD) in GCF was detected using Western blotting and immunoprecipitation with antibodies against DCD and mono-/poly-ubiquitinated proteins.	[58]
Ghosh, et al., 2010	Periodontal ligament cells	The authors used immunofluorescence, transfection assays, Western blotting, and ELISAs to show that p53 is degraded by a proteasome pathway in response to a proapoptotic disease-associated fibronectin fragment.	Investigated whether fibronectin fragments induce ubiqutination of p53 and its degradation by the proteasome. Inhibiting either the proteolytic function of the proteasome or suppressing ubiquitin at the protein level prevented degradation of p53 and subsequent apoptosis of primary periodontal ligament cells.	[59]
Li, et al., 2012	Keratocytic odontogenic tumors	They detected the expression of some key autophagy-related proteins in the clinical samples of keratocystic odontogenic tumors (KCOT) and radicular cysts and compared them via real-time quantitative polymerase chain reaction (qPCR) and immunohistochemical analysis, respectively. The correlation between the tested autophagy-related proteins with cell antiapoptotic (Bcl-2) or proliferative (Ki-67) markers in KCOT was explored using a Spearman’s rank correlation, followed by a cluster analysis.	Evaluated the activation status of autophagy in keratocystic odontogenic tumors (KCOT) and detected and compared the expression patterns of some key autophagy-related proteins in clinical samples of KCOT and radicular cysts. Implicated the activation of autophagy in KCOT and showed a possible association with growth potential.	[60]
Zeidán-Chuliá, et al., 2016	Periodontitis-associated bacteria, Human oral neutrophils	Using systems biologytools, the authors aimed to: (1) identify an integrated interactome between matrix metallo proteinase (MMP)-REDOX/nitric oxide (NO) and apoptosis pathways upstream of periodontal inflammation; and (2) characterize the attendant topological network properties to uncover putative biomarkers to be tested in the saliva of patients with periodontitis.	Found Ubiqutin C (UBC), Jun proto-oncogene (JUN), and matrix metalloproteinase-14 (MMP-14) as the most central hub- and non-hub-bottlenecks among the 211 genes/proteins of the whole interactome. Described that UBC, JUN, and MMP-14 are likely an optimal candidate group of host-derived biomarkers, in combination with oral pathogenic bacteria-derived proteins, for detecting periodontitis in its early phase.	[61]
Cai, et al., 2015	Oral epidermis tissues and epithelial cells from the White sponge nevus patients	Sequence analysis of samples from a WSN Chinese family revealed a mutation (332 T > C) in the KRT13 gene that resulted in the amino acid change Leu111Pro. The pathological pathway behind the WSN expression profile was investigated using RNA sequencing (RNA-seq).	Investigated the pathogenesis of white sponge nevus (WSN), a rare periodontal hereditary disease, by expression profiling and found that the ribosome structure was damaged and the translation rate was limited in WSN patients, while ubiquitin-mediated proteolysis was enhanced. This study concluded that the abnormal degradation of keratin 13 protein in WSN patients may be associated with keratin 7 protein and an abnormal ubiquitination process.	[63]
Jiang, et al., 2016	Periodontal ligament cells and ameliorates experimental periodontitis in rats	hPDLCs were treated with lipopolysaccharide (LPS) and pretreated with bortezomib (BTZ). mRNA and protein levels of tumor necrosis factor (TNF)-alpha, interleukin (IL)-1β, IL-6, and IL-8 were determined. The anti-inflammatory mechanism of BTZ was studied. Furthermore, experimental rat periodontitis was induced with ligature and LPS injection, and simultaneously and locally treated with BTZ (three injections/week). Four weeks after treatment, microcomputed tomography, immunohistochemistry, and histopathologic analyses were performed.	Bortezomib (BTZ) was the first proteasome inhibitor for clinical treatment of malignancies. The anti-cancer activity of BTZ is accompanied by an anti-inflammatory effect. Jiang et al., (2016) reported that in an LPS- and ligature-induced periodontal disease rat model, BTZ suppressed the expression of TNF-α, IL-1β, IL-6, and IL-8, reduced the ratio of receptor activation of RANKL/osteoprotegerin, and prevented alveolar bone resorption, suggesting that the anti-inflammatory activity of BTZ has a promising therapeutic effect against periodontal inflammatory responses in periodontal disease.	[65]
Kitagaki, et al., 2015	Periodontal ligament cells	A mouse PDL clone cell line, MPDL22, was cultured in mineralization medium in the presence or absence of bortezomib. The expression of calcification-related genes and calcified-nodule formation was evaluated by real-time PCR and Alizarin Red staining, respectively.	Investigated whether BTZ can induce differentiation of PDL cells into hard tissue-forming cells and found that BTZ enhanced the expression of bone morphogenetic protein-2, which induces cytodifferentiation and mineralization of PDL cells. BTZ induced cytodifferentiation of PDL cells by enhancing the accumulation of B-catenin within the cytosol and nucleus, suggesting that BTZ may be efficacious for use in periodontal regeneration therapy.	[66]
Yin, et al., 2007	*Fusobacterium nucleatum*, Oral epipithelial cells	Human b-defensin-2 (hBD2) is expressed in normal oral tissue leading to the hypothesis that oral epithelial cells are in an activated state with respect to innate immune responses under normal in vivo conditions. To test this hypothesis, global gene expression was evaluated in GECs in response to stimulation by an *F. nucleatum* cell wall (FnCW) preparation and hBD2 peptide. FnCW treatment altered 829 genes, while hBD2 altered 209 genes (P,0.005, ANOVA).	*F. nucleatum* and its cell wall extracts induce the expression of human beta-defensin-2 (hBD2), an antimicrobial and immunomodulatory peptide, in cultured primary human gingival epithelial cells in vitro. *F. nucleatum* cell wall extracts upregulated the expression of multiple protease inhibitors and suppressed NF-κB function and the ubiquitin/proteasome system. Both *F. nucleatum* cell wall extracts and hBD2 upregulated genes that may enhance the gingival epithelial barrier.	[67]
Shin et al., 2015	Periodontal ligament cells	To assess the apoptotic effects of STZ on periodontal ligament cells (PDLs), they were treated with or without different concentrations of STZ. Qualitative estimation of apoptotic cell death was obtained via a live/dead assay. The expression levels of apoptosis-related proteins were evaluated by a Western blot analysis.	Streptozotocin (STZ, 2-deoxy-2-3(3-(methyl-3-nitrosoureid)-d-glucopyranose) treatment dramatically reduces Mcl-1 (which induces myeloid leukemia cell differentiation protein) expression in a proteasome-dependent manner, thereby suppressing the growth of PDL cells through the Bax/Bak apoptotic signaling pathway. STZ may play an important role in inducing PDL cell apoptosis as a potential direct inducer of periodontitis in an STZ-induced diabetic animal.	[69]
